# Inflammatory bowel disease and prostate cancer: from epidemiological controversy to mechanistic hypotheses

**DOI:** 10.3389/fimmu.2026.1925968

**Published:** 2026-08-20

**Authors:** Tong Yu, Lulu Zhang, Danrui Li, Yu Wang

**Affiliations:** 1 Operating Room 1, First Hospital of Jilin University, Changchun, China; 2 Department of Hepatobiliary and Pancreatic Surgery II, First Hospital of Jilin University, Changchun, China

**Keywords:** glutamate carboxypeptidase II, gut–prostate axis, inflammation burden, inflammatory bowel disease, Mendelian randomization, prostate cancer

## Abstract

Inflammatory bowel disease (IBD) is a chronic systemic inflammatory disorder with rising global prevalence. Epidemiological evidence linking IBD to prostate cancer (PCa) risk remains conflicting. Observational cohorts suggest a possible elevated risk, though this remains uncertain and may be influenced by detection bias. Mechanistically, chronic intestinal inflammation may indirectly promote prostate carcinogenesis through systemic cytokines, immune cell infiltration, and activation of the NF-κB/STAT3/AKT signaling pathway, coupled with oxidative stress and DNA damage. The gut–prostate axis, driven by microbial dysbiosis, represents another emerging pathway. Notably, GCPII independently identified as PSMA in prostate cancer and as FOLH1 in inflamed intestinal mucosa, is upregulated in both contexts, suggesting a molecular bridge linking intestinal inflammation to prostate malignancy. However, standardized screening protocols, validated risk stratification models, and targeted prevention strategies remain lacking for the IBD population. This review synthesizes current epidemiological and mechanistic evidence, identifies surveillance bias as a primary confounder, and proposes the GCPII--glutamate--mGluR1 axis as a hypothetical yet testable mechanistic framework. We further advocate for inflammation burden-based risk stratification as a research priority, while acknowledging that this approach remains hypothetical and requires prospective validation before clinical implementation.

## 1 Introduction

Inflammatory bowel disease (IBD) is a chronic immune-mediated inflammatory disorder with rising global prevalence (1–4). IBD comprises ulcerative colitis (UC), confined to the colon, and Crohn’s disease (CD), which can affect any gastrointestinal segment with transmural inflammation (5–7). The global burden of IBD is increasing steadily, with particularly marked rises in newly industrialized countries and among children (8–10). The clinical manifestations of IBD are highly heterogeneous. Gastrointestinal symptoms primarily include chronic abdominal pain, diarrhea, tenesmus, bloating, weight loss, and fatigue (9, 11, 12). Beyond its gastrointestinal manifestations, IBD is increasingly recognized as a systemic inflammatory condition that predisposes patients to various malignancies, including colorectal cancer and certain extraintestinal tumors (13). Studies have shown that IBD-associated chronic inflammation promotes tumorigenesis by releasing large amounts of pro-inflammatory cytokines, which induce oxidative stress, DNA damage, and disruption of the immune microenvironment (14, 15). Furthermore, immunosuppressants and biologics—widely used in IBD treatment—may also influence cancer risk, adding further complexity to the disease’s systemic effects (13, 16).

Although extraintestinal complications of IBD are well recognized, accumulating evidence suggests that chronic systemic inflammation may promote malignancies in remote organs, including the prostate. Prostate cancer (PCa) is a major global health burden, with 1.47 million new cases and 0.40 million deaths in 2022, making it the second most diagnosed cancer in men worldwide (17, 18). PCa is the most common cancer in men in 112 countries and the leading cause of cancer death in 48 countries (19). PCa is strongly age-dependent, with a median diagnosis age of 67 years, and over 50% of cases occur in men aged ≥65 years (17). Over the next two decades, global incidence and mortality are expected to double, particularly among younger adults (19, 20). Given the overlapping inflammatory pathways and mounting epidemiological evidence, a comprehensive understanding of the mechanistic interface between IBD and PCa is urgently needed. In this review, we synthesize current epidemiological and mechanistic evidence to address this paradox. While acknowledging that much of the mechanistic evidence is extrapolated from other inflammatory disease models, we propose the GCPII–glutamate–mGluR1 axis as a testable mechanistic framework and, critically, leverage clinical treatment data—specifically, the effects of anti-inflammatory and immunosuppressive therapies on PCa risk—as reverse evidence to test the inflammatory burden hypothesis. We conclude by advocating for inflammation burden-based risk stratification to resolve the clinical paradox and guide precision prevention.

## 2 Literature search strategy

Given the narrative scope of this review, we did not perform a formal systematic review with a registered protocol. However, a transparent and reproducible literature search was conducted to ensure comprehensive coverage of the relevant evidence. We searched PubMed, Web of Science, and the Cochrane Library for articles published from database inception to June 2026, with the final search performed in June 2026. We applied no language restrictions, and both English and non-English publications were considered for inclusion when an English abstract was available. The search strategy combined terms related to IBD, prostate cancer, and mechanistic pathways, using the core string combining IBD terms (“inflammatory bowel disease”, “IBD”, “ulcerative colitis”, “Crohn disease”) with prostate cancer terms (“prostate cancer”, “prostate carcinoma”, “PCa”) and mechanism-related keywords (“inflammation”, “cytokine”, “gut microbiome”, “microbiota”, “dysbiosis”, “GCPII”, “PSMA”, “FOLH1”, “immune”, “metabolism”, “hormone”), and reference lists of identified articles and relevant reviews were manually screened for additional citations. We included original research articles (cohort studies, case–control studies, Mendelian randomization analyses, cross-sectional studies, animal experiments, and *in vitro* mechanistic studies), systematic reviews and meta-analyses, and relevant narrative reviews that addressed the association or mechanistic pathways between IBD and prostate cancer, while excluding case reports, conference abstracts, editorials, and studies not directly related to IBD or prostate cancer biology. For epidemiological studies, we prioritized those with adequate sample size and adjustment for key confounders, and for mechanistic studies, we included both direct evidence from the IBD–PCa setting and indirect evidence from other inflammatory or cancer models where direct evidence was limited. To facilitate interpretation of the heterogeneous evidence, we adopted an evidence-grading framework that categorizes findings into three tiers—direct evidence from human IBD–PCa cohort or case–control studies, indirect but causal evidence from animal models of colitis and prostate carcinogenesis, and plausibility evidence from *in vitro* experiments or extrapolations from other inflammation-associated cancers—which is applied transparently throughout Section 3 to flag the inferential limitations of each line of evidence and to avoid overstating the strength of the mechanistic narrative.

## 3 Recent advances in observational studies on the epidemiological association between IBD and PCa

### 3.1 Conflicting observational evidence: cohort studies from different countries and meta-analyses

Epidemiological evidence suggests that IBD patients have an increased overall cancer risk, with male reproductive system cancers notably elevated (21). Before examining mechanistic pathways, it is essential to establish the nature and strength of the epidemiological association between IBD and PCa. Based on the available evidence, three key observations emerge. First, whether IBD confers an elevated risk of PCa remains uncertain: some meta-analyses report modest associations, but these are primarily driven by UC rather than CD, and they are heavily influenced by detection-related biases. Second, the magnitude of any true association—if it exists—appears modest, with large cohort studies reporting effect sizes that are often attenuated to near-null after adjusting for screening intensity and confounders. For example, the South Korean nationwide cohort (73,805 IBD patients) reported an adjusted HR of 0.93 (95% CI: 0.68–1.27), indicating no significant difference in PCa incidence between IBD and non-IBD groups (22). Third, the distinction between UC and CD remains unresolved; while several meta-analyses suggest an association in UC but not CD, this pattern may reflect differences in surveillance intensity and disease-related healthcare utilization rather than biological distinctions. These uncertainties underscore the need to approach mechanistic hypotheses with appropriate caution and to distinguish between PCa incidence, PSA elevation, biopsy detection, and clinically significant PCa when interpreting epidemiological findings. Large-scale cohort studies from various countries have yielded contradictory conclusions regarding the epidemiological association between IBD and PCa risk, and no consensus has been reached. In the UK Biobank prospective cohort (218,084 men), Meyers et al. reported a significantly elevated risk of PCa associated with ulcerative colitis but not with Crohn’s disease (23). In contrast, a nationwide retrospective cohort study using the South Korean National Health Insurance Service database (73,805 IBD patients) found no significant difference in PCa incidence between IBD and non-IBD groups, with null results for both UC and CD (22). A single-center retrospective study in Japan observed a higher risk of PCa among UC patients, particularly those with middle-age onset and pancolitis. Notably, no PCa cases occurred among the 42 patients treated with biologics in this cohort. However, given the extremely small sample size of the biologic-treated subgroup and the limited number of events, this observation should be interpreted with caution and cannot be taken as evidence that biologic therapy reduces PCa risk; it raises a hypothesis that warrants testing in larger, adequately powered studies (24). An analysis of U.S. Veterans Affairs data (52,311 PCa patients) found that only 0.5% had coexisting IBD, mostly White, low-risk, and surgically treated. Still, this study did not provide a comparative risk estimate with non-IBD controls (25). Conversely, another U.S. matched cohort study reported a 10-year cumulative PCa incidence of 4.4% in IBD patients versus 0.65% in controls, with a markedly elevated hazard ratio of 2.44 for clinically significant PCa (26). These findings – ranging from a null effect in Korea to a more than twofold increase in the U.S. cohort – highlight substantial regional heterogeneity, suggesting that genetic background, environmental exposures, healthcare practices, and study design may profoundly influence the observed direction of the association. To synthesize the dispersed evidence, several meta-analyses have been conducted, yet their pooled estimates also diverge. A meta-analysis of 18 cohort studies (592,853 participants) reported that IBD was associated with a modestly increased risk of PCa (HR = 1.20, 95% CI: 1.06–1.37), driven primarily by UC (HR = 1.20, 95% CI: 1.06–1.38) rather than CD (HR = 1.03, 95% CI: 0.91–1.17), with the association being significant only in European populations (27). In contrast, a systematic review by Zhang et al., incorporating 16 studies, concluded that IBD did not significantly increase overall PCa risk, with only a marginal elevation observed in certain subgroups (28). The discrepancy between these meta-analyses may stem from differences in included studies, strategies for adjusting confounders, and handling of detection bias. This inconsistency at the pooled level further underscores the instability of the current observational evidence base.

Such marked inconsistency cannot be attributed solely to random variation. Among the potential drivers, detection bias is considered a core mechanism that may inflate positive findings. IBD patients, especially those with UC, undergo frequent gastroenterological follow-up and colonoscopic surveillance, which inadvertently increases opportunities for incidental PSA testing and prostate biopsy. More critically, IBD-related systemic inflammation can elevate PSA through non-malignant pathways; a causal association between UC and increased PSA concentrations has been demonstrated (29, 30). Beyond detection bias, several additional factors likely contribute to the observed heterogeneity across studies. First, disease severity and extent vary substantially among IBD cohorts. Patients with extensive colitis or more severe endoscopic activity may experience greater systemic inflammatory spillovers, potentially translating into higher PCa risk, yet most studies lack granular data on disease phenotype. Second, treatment history—particularly the use of immunosuppressants, biologics, or aminosalicylates—may modify cancer risk in opposite directions, as discussed later in Section 3.4, but is rarely accounted for in epidemiological analyses. Notably, antibiotic exposure represents an additional treatment-related confounder that has received insufficient attention. IBD patients frequently receive antibiotics for disease-related infections or complications, and antibiotic use is known to induce long-lasting alterations in gut microbiota composition. Importantly, several epidemiological studies have suggested that cumulative antibiotic exposure is associated with an increased risk of PCa, potentially through microbiota-mediated effects on systemic inflammation, immune regulation, or hormonal metabolism. This association creates a potential confounding pathway: antibiotic exposure in IBD patients may independently contribute to PCa risk, thereby complicating the interpretation of any observed association between IBD itself and PCa. However, most existing IBD–PCa cohort studies lack detailed data on antibiotic prescription history, and this limitation should be acknowledged when interpreting positive findings. Third, differences in PSA screening practices across countries and healthcare systems introduce significant ascertainment bias; for instance, the high screening intensity in the U.S. may inflate detection rates in both IBD and non-IBD groups, whereas countries with opportunistic screening may yield different patterns. Fourth, racial and ethnic composition is a critical modifier, as PCa incidence and biology differ markedly between African American, European, and Asian populations; the predominance of European-ancestry cohorts in many studies limits generalizability. Fifth, follow-up duration and latency matter considerably. IBD is frequently diagnosed in adolescence or early adulthood, whereas PCa incidence rises sharply after age 60 years, with a median diagnosis age of 67 years. This several-decade age gap means that even long-term IBD cohorts with 10-15 years of follow-up may not capture the full window of PCa risk, potentially leading to substantial underestimation of the true association. Conversely, studies with extended follow-up extending into the age range when PCa incidence peaks may be better positioned to detect a cumulative effect of chronic inflammation over decades. The substantial variability in follow-up duration across published cohort studies is therefore a major source of heterogeneity in reported risk estimates. Collectively, these intersecting factors—rather than any single confounder—likely explain the divergent findings across cohorts and underscore the need for standardized, stratified analyses in future research. In summary, the current observational evidence presents a striking paradox: some large cohorts and meta-analyses support an elevated UC-related risk, while others of comparable scale refute any association.

## 4 Triangulating evidence: from biological mechanisms to clinical reverse validation

**Figure 1:**
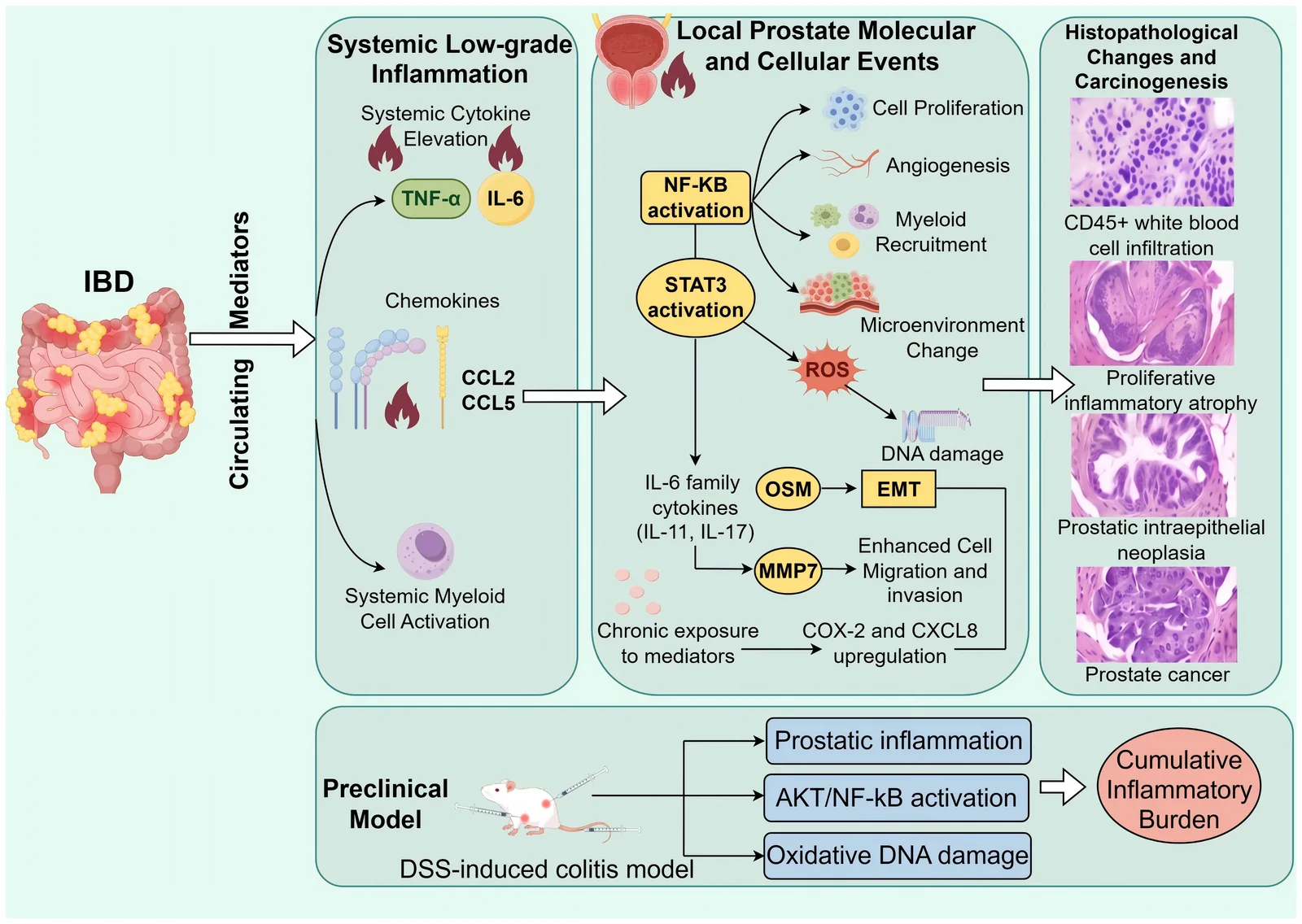
Systemic inflammatory spillover from inflamed gut to the prostate: molecular mediators, signaling cascades, and pre-neoplastic consequences. Chronic intestinal inflammation in IBD generates circulating pro-inflammatory mediators—including cytokines (TNF-α, IL-6, IL-1β, OSM, IL-17), chemokines (CCL2, CCL5, CXCL1, CXCL8), and activated myeloid cells—that disseminate systemically to the prostate. Upon arrival, these signals activate NF-κB and STAT3 signaling, driving epithelial proliferation, angiogenesis, and inflammatory reprogramming. Oxidative stress induces DNA damage, while persistent cytokine exposure sustains a feed-forward inflammatory loop. At the tissue level, these changes manifest as CD45^+^ leukocyte infiltration and proliferative inflammatory atrophy (PIA)- like lesions, precursors to prostatic intraepithelial neoplasia (PIN) and prostate cancer. This cascade is causally validated in DSS-induced colitis models and is hypothesized to scale with cumulative inflammatory burden. CCL, C-C motif chemokine ligand; COX-2, cyclooxygenase-2; CXCL, C-X-C motif chemokine ligand; DSS, dextran sodium sulfate; EMT, epithelial-mesenchymal transition; IBD, inflammatory bowel disease; IL, interleukin; MMP7, matrix metalloproteinase 7; NF-κB, nuclear factor kappa-B; OSM, oncostatin M; PCa, prostate cancer; PIA, proliferative inflammatory atrophy; PIN, prostatic intraepithelial neoplasia; ROS, reactive oxygen species; STAT3, signal transducer and activator of transcription 3; TNF-α, tumor necrosis factor-alpha.

Given the inconsistent epidemiological findings and largely negative Mendelian randomization results, the biological plausibility of an IBD–PCa link must be examined through mechanistic evidence. Chronic systemic inflammation and gut microbiome dysbiosis, two hallmarks of IBD, are interconnected candidate pathways through which intestinal inflammation may remotely influence prostate carcinogenesis. Systemic inflammation serves as a downstream executor of gut-derived immune dysregulation, while microbial metabolites sustain this inflammatory state. This section reviews how these pathways converge on shared pathogenic mechanisms in the prostate, highlighting molecular mediators, cellular effectors, and signaling cascades. Notably, GCPII, independently identified as PSMA in prostate cancer and FOLH1 in inflamed intestinal mucosa, may serve as a molecular bridge linking intestinal inflammation to prostate malignancy (**Figure 1**).

Throughout this section, we adopt a transparent evidence-grading framework to help readers distinguish observations derived directly from the IBD–PCa setting from those extrapolated from other disease contexts, categorizing the evidence into three tiers—direct evidence from human IBD–PCa cohort or case–control studies, indirect but causal evidence from animal models of colitis and prostate carcinogenesis, and plausibility evidence from *in vitro* experiments or extrapolations from other inflammation-associated cancers (e.g., colorectal cancer)—and we explicitly flag the inferential limitations of each line of evidence to ensure that the strength of the mechanistic narrative is not overstated.

It is important to emphasize that the mechanistic pathway discussed in this section—from intestinal inflammation to systemic mediators, to prostatic signaling, and ultimately to carcinogenesis—is constructed from observations derived from multiple, often separate, experimental and clinical settings. In many cases, individual links in this proposed chain have been characterized in different diseases, tissues, or model systems. Their convergence in this review serves to generate a coherent biological hypothesis, not to assert that each step has been empirically validated within the IBD–PCa axis. Wherever evidence is extrapolated from other contexts or rests on independent compartmentalized observations, we explicitly flag these inferential limitations to avoid overstating the strength of the mechanistic narrative.

### 4.1 The carcinogenic effects of chronic systemic inflammation

Despite inconclusive causal evidence from Mendelian randomization studies, the biological plausibility of an IBD–PCa link warrants mechanistic investigation. The current evidence base spans studies of widely varying inferential strength—from direct human observations and causal animal models to *in vitro* mechanistic experiments and extrapolations from other cancer contexts. This section reviews the evidence for systemic inflammation as a biological link, with explicit attention to the strength and nature of the supporting data. **Figure 1** provides an overview of this systemic spillover cascade, from gut-derived mediators to downstream prostatic signaling and pre-neoplastic consequences.

The most direct evidence for systemic spillovers of intestinal inflammation derives from both human and animal studies. IBD patients exhibit elevated circulating pro-inflammatory cytokines and activated immune cells due to persistent mucosal inflammation, which can disseminate to distant organs and establish a low-grade systemic inflammatory milieu. This systemic effect is not confined to the gut; for instance, chronic systemic inflammation in IBD has been associated with increased arterial stiffness, suggesting subclinical organ damage (**Figure 1**). In animal models, dextran sodium sulfate (DSS) induced colitis in mice, even without azoxymethane (AOM) treatment, which led to colonic tumors when combined with prostate cancer, and prostatic tissues showed histopathological changes resembling proliferative inflammatory atrophy. Concurrently, Gr1^+^ myeloid cells accumulated in the prostate microenvironment under chronic inflammatory conditions, indicating that systemic inflammation can reshape the prostate niche via circulating immune cells (31). Complementing these experimental findings, clinical data have shown that male IBD patients have a 10-year cumulative prostate cancer incidence of 4.4%, significantly higher than 0.65% in controls, and those aged over 60 years exhibit higher prostate-specific antigen (PSA) levels, further supporting that chronic intestinal inflammation may promote prostate carcinogenesis through systemic dissemination (32).

Histopathological and molecular analyses of prostate tissue from men with IBD provide additional human validation. In a landmark study, Desai et al. examined prostate specimens from IBD patients and non-IBD controls, revealing significantly increased infiltration of CD45^+^ leukocytes in IBD-associated prostate tumors, along with upregulation of pro-inflammatory factors, including TIMP-1, CCL5, and CXCL1 (15). These findings demonstrate that chronic intestinal inflammation is associated with measurable immune alterations in the remote prostate. This provides critical human validation that systemic inflammatory spillovers indeed reach the prostatic microenvironment. However, as a cross-sectional study, it cannot establish whether immune infiltration precedes or follows carcinogenesis, nor whether it is causally linked to malignant transformation. Complementing these tissue-based findings, Bhatti et al. have highlighted that systemic inflammatory biomarkers such as NLR and CRP are valuable for predicting PCa risk in patients with systemic inflammatory disorders, potentially via recruitment of tumor-associated myeloid cells and induction of an immunosuppressive microenvironment (33). Yet these correlational human data, while essential for establishing relevance, do not by themselves prove causality.

Beyond general immune cell infiltration, circulating pro-inflammatory cytokines can act directly on the prostate epithelium, as depicted in the upper portion of **Figure 1** where cytokines (TNF-α, IL-6, IL-1β, OSM, IL-17) and chemokines (CCL2, CCL5, CXCL1, CXCL8) are shown disseminating from the inflamed gut into the bloodstream and ultimately reaching the prostate. For example, oncostatin M (OSM), an IL-6 family cytokine, activates STAT3 signaling in non-transformed human prostate epithelial cells, induces epithelial–mesenchymal transition (EMT), enhances cell migration, and promotes an invasive growth pattern, suggesting that circulating inflammatory factors can directly alter prostate epithelial behavior (34). More broadly, systemic inflammation delivers pro-inflammatory cytokines (e.g., IL-6, TNF-α, IL-1β) as endocrine signals that activate proliferative pathways such as NF-κB, STAT, and HIF in prostate epithelial cells. These signals also promote the recruitment of tumor-associated myeloid cells and induce angiogenesis and vascular permeability (35). Clinical evidence further shows that serum levels of IL-1β, LY96, and TLR4 are significantly elevated in prostate cancer patients, and polymorphisms in chronic inflammation-related genes (e.g., COX-2 rs689466, NOD1 rs5743336) are closely associated with prostate cancer risk (36). In IBD patients, IL-32 can induce the expression of TNF-α, IL-6, and IL-1β, potentially contributing to disease progression through systemic inflammatory effects, although its specific role in prostate cancer remains to be defined; its pro-inflammatory properties nonetheless suggest a plausible pathogenic link.

Causal validation in animal models. To establish whether intestinal inflammation can causally induce prostatic changes, animal experiments have provided critical mechanistic validation. Using the dextran sodium sulfate (DSS)-induced colitis model in mice, Desai et al. demonstrated that chronic colitis leads to CD45^+^ leukocyte infiltration into prostate tissue, upregulation of pro-inflammatory factors, activation of AKT/NF-κB pathways, and evidence of oxidative stress and DNA damage in prostatic epithelial cells (15). However, the observations mentioned above reflect changes related to inflammation and precancerous lesions, not an increase in diagnosed invasive cancer or cancer incidence. This study didn’t show that colitis raises the risk of prostate cancer or causes it to progress to invasive malignant tumors. These findings causally link intestinal inflammation to remote prostate changes in the absence of confounding factors and surveillance biases that plague human studies. This model recapitulates the systemic inflammatory spillover hypothesis: inflammatory cells migrate from the inflamed gut to the prostate via the bloodstream, where chemokines recruit monocytes and mast cells, remodeling the local microenvironment toward a pro-tumorigenic state. However, the DSS model represents acute chemical injury rather than the chronic immune-mediated inflammation characteristic of human IBD. Moreover, the observed changes are inflammatory and pre-neoplastic rather than frankly malignant. Whether these changes progress to invasive carcinoma without additional oncogenic hits remains to be established.

Chronic inflammation also drives genomic instability through oxidative stress and excessive production of reactive oxygen species (ROS), which attack DNA bases, increase mutation frequency, and interfere with DNA repair mechanisms (15). Proliferative inflammatory atrophy (PIA) is recognized as an intermediate pre-neoplastic lesion in the prostate; its luminal epithelial cells are prone to genetic alterations and may progress to prostatic intraepithelial neoplasia (PIN) and eventually prostate cancer. Animal experiments have confirmed that inflammation can reprogram prostate epithelial cells, thereby constituting a key step in tumor initiation, and that prostate infection or epithelial barrier disruption may drive the establishment of a persistent inflammatory microenvironment, with the urinary microbiome potentially contributing to long-term microbial exposure (15). In colitis-associated cancer (CAC) models, inhibition of IL-1β reduces cancer stem cell formation and tumor burden, underscoring the role of inflammatory cytokines in maintaining genomic instability and stemness (18). Although not fully elucidated in prostate cancer, the pathophysiological logic—chronic inflammation driving repeated epithelial injury and aberrant repair, leading to tissue remodeling and genomic instability—is well validated in IBD-associated colorectal cancer and shares substantial mechanistic parallels with prostate carcinogenesis (37).

Molecular mechanisms from *in vitro* and extrapolated models. The pathways through which systemic inflammation may promote prostate carcinogenesis have been extensively characterized in cell culture systems and extrapolated from other inflammatory cancer contexts. Chronic inflammation activates transcription factors such as NF-κB and STAT3—a cascade illustrated in the central portion of **Figure 1**—driving the sustained release of pro-inflammatory cytokines, including TNF-α, IL-6, and IL-1β, which in turn promote angiogenesis, tissue destruction, and tumor progression (38).In prostate epithelial cell lines, IL-6 family cytokines, such as oncostatin M, can induce epithelial-mesenchymal transition (EMT) and enhance cell invasion, effects mediated in part by STAT3 signaling (38). IL-17 has also been shown to mediate EMT via MMP7 in prostate cancer cell models (39). Beyond these cytokine-driven effects, *in vitro* studies using benign prostatic hyperplasia epithelial cells infected with Trichomonas vaginalis demonstrated that infected cells release CCL2, IL-1β, IL-6, and CXCL8, thereby inducing mast cell and monocyte migration while promoting stromal proliferation through the CXCL8–CXCR1 and CCL2–CCR2 pathways, accompanied by upregulation of FGF2, cyclin D1, and Bcl-2 (33). Furthermore, chronic exposure to IL-1 can attenuate the synergistic cell-cycle-inhibitory effects normally exerted by combined IL-1 and IL-6 stimulation, suggesting that prolonged inflammatory stimulation reprograms cellular responses to signaling pathways (39). Tumor-associated macrophages and myeloid-derived suppressor cells have also been implicated in inflammation-associated prostate cancer, further underscoring the complexity of immune–stromal crosstalk in the tumor microenvironment (39). While these *in vitro* findings identify a range of plausible molecular mechanisms—including activation of the NF-κB/STAT3/AKT pathway, upregulation of COX-2 and CXCL8, and induction of EMT—they are derived from cell lines that may not fully recapitulate the complexity of the tumor microenvironment. Moreover, the cytokine concentrations used in many experiments often exceed physiologically relevant levels. Moreover, many of these pathways were initially characterized in other inflammatory cancer models and subsequently extrapolated to prostate cancer, rather than being directly validated within the specific IBD–prostate axis.

A caveat must be noted here: the signaling pathways discussed—including NF-κB, STAT3, and AKT—are not specific to the IBD–PCa axis. They represent shared mechanisms of inflammation-associated carcinogenesis that operate across many tissue types and disease contexts. Their individual involvement in IBD and in prostate cancer supports biological plausibility but does not, by itself, establish an IBD-specific causal pathway. The same caution applies to cytokines such as IL-6, TNF-α, and IL-1β, which are ubiquitous mediators of inflammatory responses.

The strongest causal evidence for systemic inflammatory spillover comes from animal models (DSS-induced colitis), which demonstrate that intestinal inflammation can remotely induce prostatic inflammatory and pre-neoplastic changes. Human data from prostate tissue analyses confirm immune alterations in IBD patients but remain cross-sectional and cannot establish temporality. The detailed molecular pathways, including EMT, specific cytokine cascades, and DNA repair interference, are largely derived from *in vitro* studies and extrapolations from colorectal cancer models. While biologically plausible, their direct relevance to the IBD–PCa axis requires experimental confirmation in disease-specific models.

### 4.2 Gut microbiome dysbiosis and the gut–prostate axis

**Figure 2:**
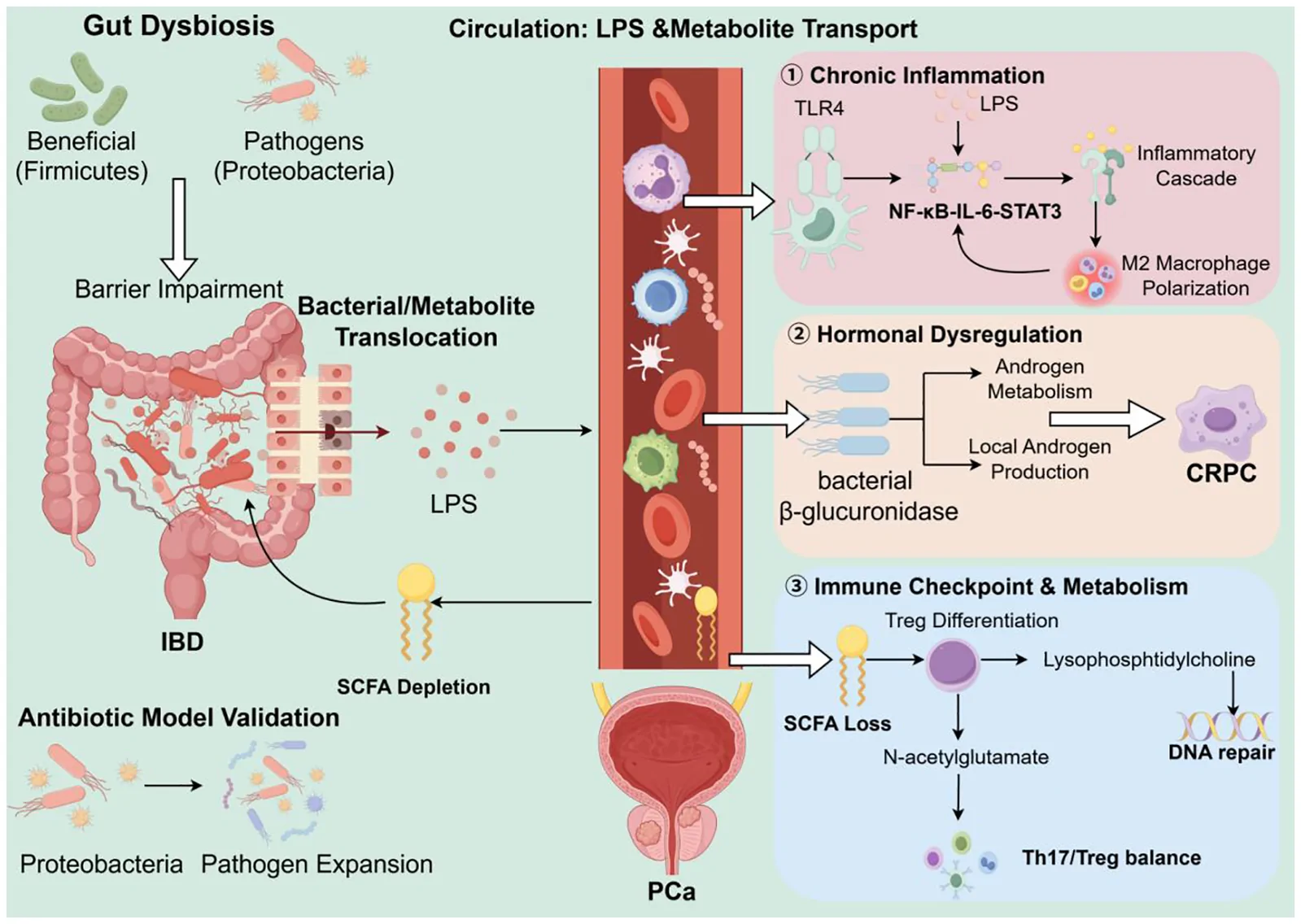
The gut–prostate axis: microbial dysbiosis, metabolite shifts, and remote microenvironmental remodeling in prostate carcinogenesis. IBD-associated gut microbiome dysbiosis—characterized by reduced α-diversity, depletion of beneficial taxa (Firmicutes, Bacteroidetes), and expansion of facultative anaerobes such as Proteobacteria—triggers three interconnected pathways that remotely influence the prostate. First, diminished SCFA production compromises epithelial barrier integrity, facilitating LPS translocation into the circulation. Second, systemic LPS activates prostatic TLR4 and the NF-κB–IL-6–STAT3 axis, promoting M2-type macrophage polarization and a tumor-permissive microenvironment. Third, microbial metabolites modulate androgen metabolism, Treg/Th17 balance, and DNA repair. Fecal Proteobacteria abundance correlates with serum IL-6 and metastasis in PCa patients, and FMT studies demonstrate that dysbiotic microbiota can accelerate prostate cancer progression. CRPC, castration-resistant prostate cancer; FMT, fecal microbiota transplantation; IBD, inflammatory bowel disease; IL, interleukin; LPS, lipopolysaccharide; NF-κB, nuclear factor kappa-B; PCa, prostate cancer; SCFA, short-chain fatty acid; STAT3, signal transducer and activator of transcription 3; Th17, T helper 17; TLR4, Toll-like receptor 4; Treg, regulatory T cell.

Gut microbiome dysbiosis, a hallmark of chronic intestinal inflammation, is increasingly recognized as a systemic modulator of prostate homeostasis via the gut–prostate axis (**Figure 2**). Before reviewing the evidence, it is important to acknowledge that the current literature on the gut–prostate axis comprises studies of substantially different relevance to the IBD population. Direct evidence from IBD patients linking gut microbial alterations to subsequent PCa risk is extremely limited. Most mechanistic insights derive from animal models of colitis or from prostate cancer cohorts that did not specifically include IBD patients. Throughout this subsection, we explicitly distinguish observations drawn directly from IBD cohorts from those extrapolated from other settings, and we flag these inferential distinctions to avoid overstating the strength of the evidence for an IBD-specific gut–prostate axis. A critical interpretive note is warranted before reviewing the evidence on the gut–prostate axis. The studies discussed in this subsection derive from substantially different sources: observational profiling of the gut microbiota in IBD patients; cross-sectional comparisons of microbial composition in men with established PCa; antibiotic-induced dysbiosis models in mice; fecal microbiota transplantation experiments; and mechanistic inferences from colorectal cancer or benign prostatic inflammation. These lines of evidence, while individually informative, cannot be concatenated to demonstrate that IBD-associated dysbiosis precedes and causes PCa in humans. At best, they provide indirect support for biological plausibility; at worst, they risk creating an illusion of causal continuity where none exists. Through multiple interconnected pathways, microbial imbalance can remotely influence prostate carcinogenesis and progression. In IBD patients, gut microbiota disruption exhibits distinctive features: reduced α-diversity, depletion of beneficial obligate anaerobes (*Firmicutes*, *Bacteroidetes*, and *Actinobacteria*), and marked expansion of facultative anaerobes, such as Proteobacteria and opportunistic Enterobacteriaceae (31, 34, 35). This structural imbalance not only disrupts intestinal homeostasis but also alters key metabolic functions, leading to decreased levels of immunoregulatory and barrier-protective metabolites—including short-chain fatty acids (SCFAs), tryptophan derivatives, and secondary bile acids—alongside elevated pro-inflammatory metabolites (40, 41). Animal models confirm that genetic defects, such as Muc2 mutation, can induce early dysbiosis characterized by increased *Bacteroidetes* even before histological inflammation develops (40). Global burden of disease data further show that IBD-endemic regions exhibit ~40% reductions in beneficial bacteria, ~60% increases in harmful bacteria, ~10% decreases in Shannon diversity, and a ~20% decline in SCFAs, which correlates with a 25% increase in disability-adjusted life years (36). These compositional and functional alterations collectively compromise intestinal barrier integrity, exacerbate mucosal immune imbalance, and establish the pathological foundation for subsequent systemic effects (42, 43). A primary mechanism involves reduced production of short-chain fatty acids (SCFAs) due to microbial depletion, which compromises epithelial barrier integrity and facilitates the translocation of bacterial components, particularly lipopolysaccharide (LPS), into the circulation (44). IBD-associated barrier dysfunction can promote translocation of gut bacteria and their products through the damaged epithelial layer into the portal vein or the lymphatic system, thereby activating immune responses in distant organs (45). In addition to the direct effects of bacterial translocation, IBD-associated barrier dysfunction—resulting from reduced SCFA production and disrupted tight junctions—may facilitate the systemic dissemination of LPS and other bacterial products. Although this mechanism has been well documented in animal models of colitis and sepsis, direct evidence linking IBD-related LPS translocation to subsequent prostatic inflammation in humans remains limited. Animal experiments have shown that antibiotic-induced dysbiosis, characterized by Proteobacteria enrichment, significantly increases gut permeability and intraprostatic LPS levels, thereby mechanistically activating the NF-κB-IL-6-STAT3 signaling axis, which promotes prostate cancer growth and docetaxel resistance (45).While these findings experimentally validate that antibiotic-induced microbiota disruption can promote prostate tumorigenesis, it is important to recognize that therapeutic antibiotic exposure in IBD patients—beyond its role in experimental models—may itself represent a confounding factor in observational studies of IBD–PCa association. Clinically, patients with metastatic prostate cancer exhibit significantly higher fecal Proteobacteria abundance, which positively correlates with serum IL-6, regional lymph node metastasis, and distant metastasis (45). Furthermore, bacterial translocation can continuously activate Toll-like receptors (TLRs), sustain MAPK and PI3K pathways, and promote M2-type macrophage polarization, collectively fostering a chronic inflammatory microenvironment conducive to tumor progression (46). Although direct clinical evidence linking bacterial translocation in IBD patients to subsequent prostate cancer remains lacking, these mechanisms provide a crucial pathophysiological bridge for the “gut-prostate axis” (31, 47, 48). As summarized in the central pathway of **Figure 2**, systemic LPS then upregulates prostatic TLR4, promotes M2-type macrophage polarization, and activates IGF-1 signaling, collectively remodeling the microenvironment toward a tumor-permissive state (46). Critically, in antibiotic-induced dysbiosis models, enrichment of Proteobacteria further increases gut permeability and intraprostatic LPS levels, mechanistically triggering the NF-κB–IL-6–STAT3 axis, which drives tumor growth and docetaxel resistance; clinically, fecal Proteobacteria abundance in prostate cancer patients positively correlates with plasma IL-6, regional lymph node metastasis, and distant metastasis, and its predictive performance for distant metastasis even surpasses that of PSA (45).

PCa patients exhibit significant shifts in gut microbial communities, with genera such as *Odoribacter* and *Desulfovibrio* associated with disease status (49). Moreover, certain intestinal bacteria possess enzymes such as β-glucuronidase that participate in androgen metabolism, thereby influencing systemic hormone levels and tumor progression (50). Beyond direct enzymatic actions, gut microbial metabolites play critical roles in regulating systemic immunity, hormonal balance, and epigenetic modifications, which may directly impact prostate carcinogenesis (51, 52). SCFAs (e.g., butyrate, propionate) are major fermentation products with anti-inflammatory properties, barrier-maintaining capacity, and the ability to induce regulatory T-cell (Treg) differentiation; their significant reduction in IBD patients may lead to immune dyshomeostasis and indirectly promote tumorigenesis (38) (50). Additionally, the microbiota contributes to androgen metabolism, and specific microbial profiles may sustain local androgen production, thereby influencing the progression of castration-resistant prostate cancer (CRPC) (51). In a fecal microbiota transplantation (FMT) study, transferring CRPC patient-derived microbiota into TRAMP mice accelerated prostate cancer progression, accompanied by elevated fecal lysophosphatidylcholine and phosphatidylcholine levels, and upregulated prostatic expression of LPCAT1, RAD51, and DNA-PKcs, suggesting that the microbiota participates in carcinogenesis through regulating phospholipid metabolism and DNA repair pathways (53). Furthermore, decreased fecal N-acetylglutamate levels in IBD patients—a metabolite that can inhibit pathogenic Th17 cell differentiation and promote Treg generation—may exacerbate systemic inflammation (54). Metabolites such as taurine and betaine have also been linked to IBD risk in Mendelian randomization studies, suggesting their potential roles in inflammation-driven tumorigenesis (37). Although direct evidence remains limited, existing data strongly support the role of microbial metabolites in prostate cancer regulation through immune modulation, hormonal metabolism, and genomic stability pathways (40, 52, 55). Beyond LPS, gut microbial metabolites—including secondary bile acids, tryptophan derivatives, and short-chain fatty acids—have been implicated in regulating systemic immunity and androgen metabolism. For instance, secondary bile acids can modulate the activity of the farnesoid X receptor (FXR) and influence intestinal barrier integrity, while altered tryptophan metabolism may affect systemic kynurenine levels and immune tolerance. However, the specific contribution of these metabolite changes in IBD patients to prostate carcinogenesis remains speculative. The urogenital microbiome also displays distinct profiles correlated with Gleason score, suggesting that both local and distal microbial ecosystems contribute to remodeling of the prostatic microenvironment (56). Collectively, these observations support the gut–prostate axis hypothesis, wherein dysbiosis affects prostate malignancy through metabolic leakage, immune activation, and hormonal modulation (51, 57). The existing evidence spans a wide inferential spectrum: animal models provide causal support for the gut–prostate axis, cross-sectional studies identify compositional associations without temporal directionality, but prospective human data linking pre-diagnostic gut microbiome profiles to incident PCa remain conspicuously absent. Thus, while biologically plausible, the translational bridge to human IBD–PCa risk prediction remains incomplete, warranting prospective longitudinal multi-omics sampling, particularly in high-risk IBD populations. In patients with IBD, gut microbiota disruption is a core feature and may exacerbate systemic inflammation and immune dysfunction, potentially increasing prostate cancer risk (58). Mechanistically, the microbiota regulates IL-23 receptor-positive cells (mainly ILC3s and T cells); upon IL-23 stimulation, ILC3s upregulate CTLA-4 in a FOXO1- and STAT3-dependent manner, modulating co-stimulatory molecules on intestinal myeloid cells and PD-L1 bioavailability, thereby influencing both local and systemic immune homeostasis (50). In human IBD, ILC3-derived CTLA-4 is upregulated in response to intestinal inflammation, underscoring the importance of microbiota–immune crosstalk in maintaining inflammatory balance.

Beyond LPS, other microbial metabolites, including secondary bile acids and bacterial genotoxins, as shown in the lower portion of **Figure 2**, can modulate androgen metabolism and interfere with DNA repair, thereby contributing to genomic instability (44). Preclinical evidence indicates that dietary interventions, probiotics, or fecal microbiota transplantation can restore microbial balance, suppress tumor growth, and improve therapeutic responses (59). For instance, a mango polyphenol intervention in IBD patients increased Lactobacillus abundance and butyrate levels while reducing inflammatory cytokines such as IL-8 and GRO, indirectly suggesting that microbiota modulation may confer beneficial effects on prostate health (60). However, it must be emphasized that the extrapolation from colorectal cancer models to prostate cancer, for example, the hypothesis that high-fat diet-induced intestinal oxidative stress generates 4-hydroxynonenal, which may create a pro-tumorigenic microenvironment, remains entirely speculative for the prostate, as this effect has been documented mainly in colorectal cancer and has not been directly tested in prostate-specific models (56).In patients with IBD, gut microbiota disruption is a core feature and may exacerbate systemic inflammation and immune dysfunction, potentially increasing prostate cancer risk (58).

A less explored but potentially relevant pathway involves altered gut motility in IBD, which may affect the composition and metabolic activity of the microbiota, as well as the transit time of luminal contents and the exposure of the mucosal immune system to bacterial antigens. While chronic constipation and altered transit have been associated with changes in the gut microbiome composition, their direct relevance to systemic inflammation and prostate cancer risk remains entirely hypothetical and requires further investigation. In summary, the evidence linking gut microbiome dysbiosis to prostate cancer can be organized into three distinct tiers of relevance to the IBD population. The strongest—yet most limited—line of evidence comes from animal models, including antibiotic-induced dysbiosis and fecal microbiota transplantation studies, which demonstrate that microbial alterations can drive prostate tumorigenesis and therapy resistance. However, these models do not fully recapitulate the chronic, immune-mediated inflammation of human IBD. An intermediate line of evidence derives from cross-sectional studies in PCa patients without IBD, which have identified compositional shifts and metabolite alterations associated with disease status, but these designs cannot establish temporality or causation, and their findings cannot be directly extrapolated to IBD populations. The weakest link—and the most critical gap—is the complete absence of prospective human data examining whether pre-diagnostic gut microbiome profiles in IBD patients predict subsequent PCa risk. Moreover, many proposed mechanisms—particularly those involving androgen metabolism and DNA repair via microbial metabolites—are inferred from correlational studies or colorectal cancer models and require direct validation in IBD-specific longitudinal settings. Thus, while the gut–prostate axis is biologically plausible and supported by indirect evidence, the translational gap to human IBD remains wide, and the axis remains unproven in the IBD-specific context. This distinction must be kept in mind when interpreting the mechanistic model proposed in **Figure 2**.

### 4.3 GCPII as a potential molecular bridge between intestinal inflammation and prostate malignancy

**Figure 3:**
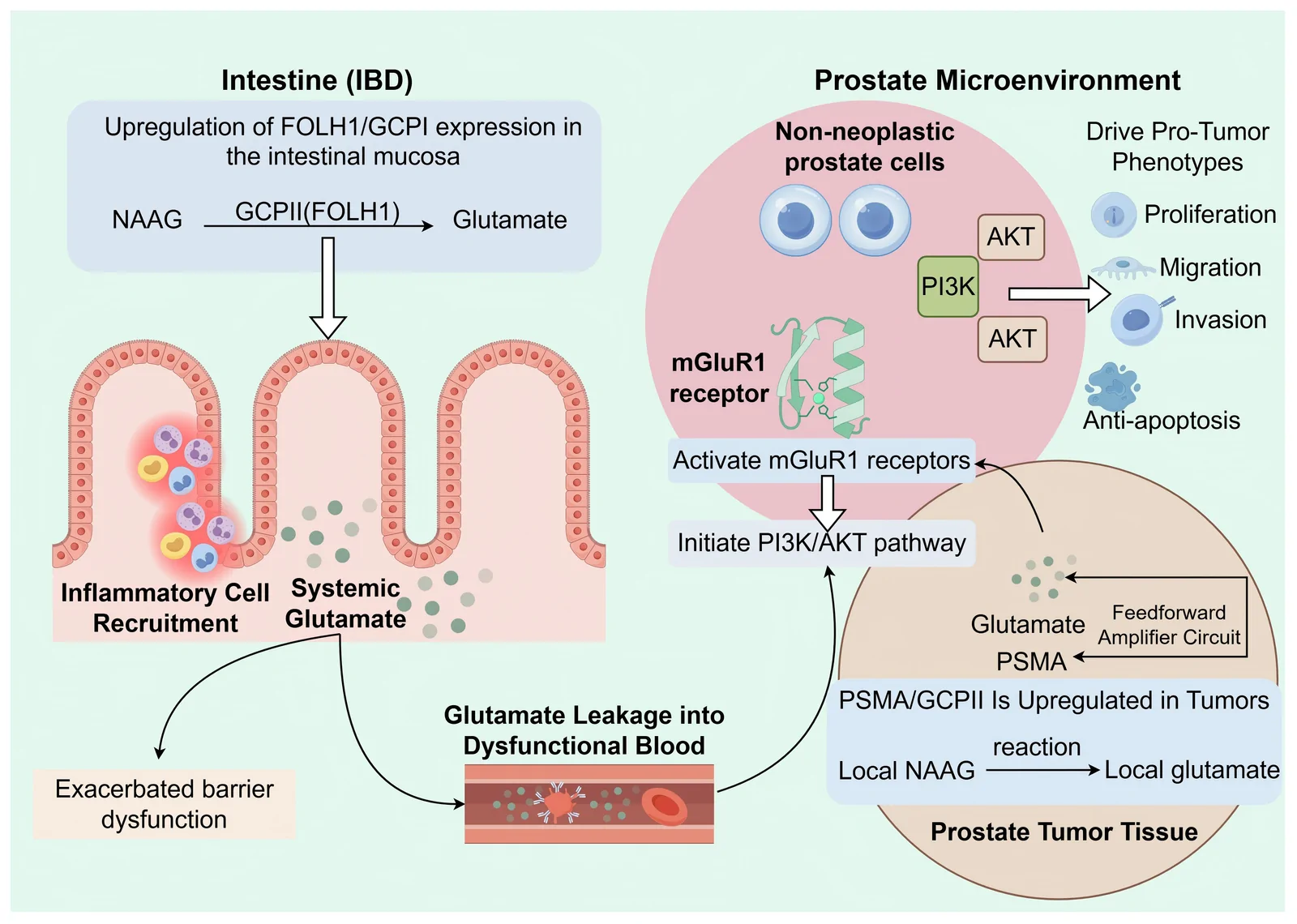
The GCPII–Glutamate–mGluR1 axis as a shared molecular bridge linking intestinal FOLH1 upregulation to prostatic PSMA-Driven carcinogenesis. Glutamate carboxypeptidase II (GCPII), encoded by the FOLH1 gene, is a type II transmembrane metallopeptidase known by three tissue-specific names: PSMA in the prostate, FOLH1 in the intestine, and NAALADase in the brain—all products of the same gene. In IBD, FOLH1 is upregulated in inflamed mucosa and functionally drives intestinal inflammation. In prostate cancer, PSMA is overexpressed on malignant epithelial cells, where its cleavage of N-acetylaspartylglutamate (NAAG) releases glutamate, which activates mGluR1 and PI3K/AKT signaling to promote proliferation and invasion. Serum glutamate correlates positively with Gleason score. Whether intestinally derived glutamate contributes to this circulating pool and primes the prostate for transformation remains a testable hypothesis. A critical knowledge gap is whether the same IBD patient who later develops PCa exhibits both intestinal FOLH1 and prostatic PSMA overexpression in a temporally coordinated manner. PSMA-PET imaging offers a potential approach to test this hypothesis. FOLH1, folate hydrolase 1; GCPII, glutamate carboxypeptidase II; IBD, inflammatory bowel disease; mGluR1, metabotropic glutamate receptor 1; NAAG, N-acetylaspartylglutamate; NAALADase, N-acetylated-alpha-linked acidic dipeptidase; PCa, prostate cancer; PET, positron emission tomography; PI3K, phosphoinositide 3-kinase; PSMA, prostate-specific membrane antigen.

Beyond systemic inflammation and gut dysbiosis, glutamate carboxypeptidase II (GCPII)—encoded by the FOLH1 gene—is a compelling molecular candidate that may functionally link intestinal inflammation to prostate carcinogenesis (61, 62). GCPII is a type II transmembrane metallopeptidase known by three names reflecting its tissue-specific discovery: PSMA in the prostate, FOLH1 in the intestine, and NAALADase in the brain—all encoded by the same gene (62). Throughout this section, we use the following naming conventions: PSMA for prostate cancer biology, FOLH1 for intestinal inflammation, and GCPII for the enzymatic function or the protein in general contexts (**Figure 3**). This convergence across distinct disease fields points to a shared molecular apparatus that may operate in both intestinal inflammation and prostate malignancy. However, this convergence remains a theoretical hypothesis rather than an empirically validated pathway. The evidence reviewed in this section is drawn from independent observations in separate disease contexts—FOLH1 upregulation in IBD intestinal mucosa and PSMA upregulation in prostate cancer—and no study has yet integrated these findings within the same individuals or demonstrated a functional link between intestinal GCPII activity and prostate carcinogenesis. Readers should therefore interpret the following discussion as a hypothesis-generating framework that requires rigorous experimental testing, rather than as an established pathogenic mechanism. The most critical gap in this proposed model is the absence of evidence linking intestinally derived glutamate to prostatic signaling at biologically relevant concentrations. While GCPII can hydrolyze NAAG to release glutamate in both intestinal and prostate tissues, no study has demonstrated that glutamate generated in the inflamed intestine reaches the prostate in sufficient quantities to activate mGluR1-mediated signaling or to induce malignant transformation. This fundamental gap must be kept in mind when considering the GCPII axis as a potential molecular bridge.

In prostate cancer, GCPII/PSMA is markedly upregulated in malignant versus benign tissue (63, 64), and its enzymatic cleavage of N-acetylaspartylglutamate (NAAG) releases glutamate, which activates metabotropic glutamate receptor (mGluR) signaling to promote proliferation, migration, and invasiveness; functional studies confirm that GCPII knockdown induces cell-cycle arrest and reduces invasiveness (65, 66). Concurrently, in IBD, FOLH1 is strongly upregulated in the inflamed intestinal mucosa, with enzymatic activity increasing 2.8- to 41-fold in affected tissues (61). Pharmacological inhibition of GCPII or FOLH1 knockout substantially ameliorates colitis in murine models, establishing that GCPII activity is a functional driver of intestinal inflammation rather than a passive marker (61). However, these observations are entirely compartmentalized: FOLH1 upregulation has been documented in IBD intestinal mucosa, and PSMA upregulation has been documented in prostate cancer, but these findings come from independent studies conducted in different patient populations, and no investigation has examined intra-individual coordination or demonstrated that the same IBD patient who later develops prostate cancer exhibits both phenomena in a temporally linked manner.

However, a critical knowledge gap remains: the current evidence is fundamentally cross-sectional and compartmentalized. Studies have independently reported upregulation of FOLH1 in the gut mucosa of inflammatory bowel disease and upregulation of PSMA in prostate cancer. As shown in the left and right panels of **Figure 3**, there hasn’t been any systematic research coordinating individuals yet. Whether the same IBD patient who later develops prostate cancer exhibits both intestinal FOLH1 overexpression and prostatic PSMA overexpression in a temporally linked manner. Establishing such co-localization would transform GCPII from a coincidental biomarker into a credible mediator of comorbidity. Notably, the wide clinical use of PSMA-PET imaging in prostate cancer has documented GCPII upregulation in various tumors and inflammatory diseases, likely associated with angiogenesis (62). Recent studies further demonstrate that PSMA-targeted PET radiotracers can effectively detect regions of inflamed mucosa in IBD patients, raising the testable possibility that serial PSMA-PET imaging in IBD patients could provide a non-invasive window into both intestinal FOLH1 expression and prostatic PSMA expression within the same individual over time, enabling the first direct test of the intra-individual co-localization hypothesis (62).

From NAAG to glutamate: a functional connection. The enzymatic function of GCPII—cleaving NAAG to release glutamate—provides the molecular link between the two disease contexts at the pathway level. In prostate cancer, glutamate activates mGluR1, which in turn stimulates the PI3K/AKT pathway signaling cascade outlined in the right panel of **Figure 3**. The same signaling node is engaged by IBD-derived systemic cytokines (65). NAAG has been identified as a glutamate reservoir in cancers expressing GCPII, and pharmacological inhibition of GCPII reduces both intracellular glutamate concentrations and tumor growth, thereby establishing that GCPII-mediated NAAG hydrolysis is a quantitatively significant source of glutamate (67). Serum glutamate levels correlate positively with Gleason score in patients with prostate cancer, and glutamate deprivation or mGluR1 receptor blockade reduces prostate cancer cell proliferation, migration, and invasion while inducing apoptosis (67). These observations establish that circulating glutamate—regardless of its origin—is biologically active in the prostate microenvironment. Whether intestinally derived glutamate contributes measurably to this circulating pool remains to be directly tested. Nevertheless, a coherent mechanistic rationale emerges from three convergent observations: elevated mucosal GCPII activity, the documented capacity of NAAG hydrolysis to generate glutamate, and the demonstrated bioactivity of glutamate in prostate cancer cells. In IBD, emerging evidence indicates that glutamate also acts as a pro-inflammatory mediator in the intestinal mucosa, contributing to epithelial barrier disruption, immune cell recruitment, and fibrosis (61). Thus, GCPII upregulation in IBD may serve a dual pathogenic function. It could sustain local intestinal inflammation while also releasing glutamate into the circulation, thereby creating a systemic glutamatergic signal that may remotely prime the prostate for neoplastic transformation. However, this concept currently remains a testable hypothesis, as direct evidence linking intestinally derived glutamate to prostatic signaling in the same individual is entirely lacking. Future studies employing isotope-labeled glutamate tracing in IBD models are urgently needed to explore this intriguing possibility.

In summary, the GCPII--glutamate--mGluR1 axis is conceptually intriguing and mechanistically plausible, but it is also the least directly validated component of the IBD–PCa mechanistic framework. The upregulation of FOLH1 in IBD mucosa and PSMA in PCa has been documented independently, yet no study has examined their intra-individual temporal co-expression or demonstrated a causal link between intestinal GCPII activity and prostate carcinogenesis. The glutamate–mGluR1–PI3K/AKT cascade has been characterized in prostate cancer cell lines, but its activation by intestinally derived glutamate remains entirely speculative. Thus, while this axis provides a coherent hypothesis for future investigation, it currently rests on indirect evidence and extrapolation from separate disease silos, rather than on integrated human or animal data. The hypothesis should be considered unproven, and its exploration in future research should be prioritized accordingly.

### 4.4 Pharmacoepidemiologic observations compatible with the inflammatory burden hypothesis

If chronic systemic inflammation is indeed a driver of prostate carcinogenesis, one might hypothesize that therapeutic interventions reducing inflammatory burden would lower PCa risk, whereas treatments that suppress immune surveillance might increase it. This section reviews pharmacoepidemiologic observations from IBD treatment studies—specifically the divergent effects of anti-inflammatory and immunosuppressive agents—as indirect evidence that may be compatible with, but cannot validate, the inflammatory burden hypothesis. It is important to emphasize that these observations derive from non-randomized, observational studies and are subject to substantial confounding, including confounding by indication, treatment selection, disease severity, adherence, healthcare utilization, and residual confounders. They should therefore be interpreted as hypothesis-generating rather than hypothesis-testing.

#### 4.4.1 Immunosuppressants and biologics: theoretical risk versus empirical evidence

Patients with IBD often require long-term treatment with immunosuppressants or biologics to control intestinal inflammation; whether such treatments increase the risk of PCa has long been a focus of clinical concern. Current evidence suggests that immunosuppressive therapy may, in theory, increase the risk of malignant tumor development by weakening the body’s immune surveillance function (68–70). However, specific data on prostate cancer remains limited. A prospective cohort study involving 305 IBD patients with a history of cancer found no statistically significant association between immunosuppressant treatment and subsequent new or recurrent cancers (71). Another large retrospective cohort study showed that, among IBD patients with a history of non-gastrointestinal malignancies, the use of biologics such as anti-TNF agents or vedolizumab did not significantly increase subsequent cancer incidence (72). A recent cohort study comparing vedolizumab, ustekinumab, and anti-TNF therapy on new-onset cancer found no significant differences (73). Nevertheless, some studies suggest that potential risks should be carefully evaluated; a systematic review noted that azathioprine and anti-TNF agents are associated with an increased risk of new or recurrent cancer, though evidence is primarily from registry and observational data lacking randomized controlled trial support (74). It must be emphasized that the absence of an observed increase in PCa risk during immunosuppressive treatment does not constitute evidence that controlling inflammation prevents PCa. Similarly, the observation of zero PCa events in the small biologic-treated subgroup of the Japanese cohort (24)—comprising only 42 patients—cannot be interpreted as evidence of a protective effect, given the extremely limited statistical power and the absence of adjustment for confounding factors such as disease severity, screening intensity, and baseline risk.

#### 4.4.2 Anti-inflammatory therapy: a protective signal

In contrast, some anti-inflammatory drugs may exert a protective effect against PCa by inhibiting chronic inflammatory pathways. A cohort study using the UK Clinical Practice Research Datalink (CPRD) reported an association between aminosalicylate exposure and reduced PCa risk among IBD patients, with an adjusted odds ratio of 0.32 (75). While this observation is intriguing, it is based on a non-randomized pharmacoepidemiologic study and remains vulnerable to multiple sources of bias, including confounding by indication, treatment selection, adherence, disease phenotype, and healthcare utilization. Whether this association reflects a genuine protective effect of 5-ASA, a marker of milder underlying disease, or residual confounding cannot be determined from these data alone. This observation is compatible with the inflammatory burden hypothesis but provides no confirmatory evidence. However, this protective effect has not been widely validated. Most epidemiological studies on IBD–PCa association have not specifically distinguished the effects of different drug regimens on cancer risk (27, 76–79). Furthermore, some studies observed no significant PCa risk increase among IBD patients—for instance, a South Korean cohort study using the National Health Insurance Service database showed no significant difference in PCa risk between IBD patients and the non-IBD population (22), which further undermines the hypothesis of a universal protective effect of anti-inflammatory treatment. It is worth noting that androgen deprivation therapy (ADT), a standard PCa treatment, has conversely been found to potentially reduce the risk of developing UC (37), indirectly suggesting complex interactions between hormonal and inflammatory pathways.

However, several caveats must be considered when interpreting the apparent protective association between 5-ASA and PCa risk. First, in clinical practice, 5-ASA is primarily prescribed for patients with mild-to-moderate IBD, whereas patients with more severe or refractory disease are more likely to receive immunosuppressants or biologics. Therefore, the lower PCa risk observed in the 5-ASA group may partly reflect differences in baseline disease severity rather than a direct pharmacological effect of the drug. Patients with milder disease inherently carry a lower cumulative inflammatory burden, which, under the inflammatory burden hypothesis, would translate into reduced PCa risk regardless of medication. This confounding by indication is a major limitation of observational drug–cancer studies and cannot be fully addressed without randomized data or rigorous adjustment for disease activity over time. Second, beyond its anti-inflammatory properties, 5-ASA has been shown to exert direct antiproliferative and pro-apoptotic effects in various cancer cell lines, including colorectal cancer cell lines, through mechanisms such as inhibition of cyclooxygenase-2 (COX-2), modulation of the Wnt/β-catenin pathway, and induction of cell-cycle arrest. Whether such direct tumor-suppressive effects extend to prostate cancer cells remains largely unexplored, but this possibility warrants further investigation. Thus, while the 5-ASA signal is intriguing, it may arise from a combination of confounding by disease severity and genuine anti-tumor activity—effects that current epidemiological studies cannot reliably disentangle.

#### 4.4.3 Integrating the evidence: what do these clinical data tell us

When synthesized, these pharmacological observations offer intriguing, albeit circumstantial, clues that align with the inflammatory burden hypothesis. For instance, the potential protective signal associated with 5-ASA and the null effect of biologics on PCa risk in some cohorts are suggestive but far from conclusive. These interpretations remain heavily constrained by confounding by indication, channeling bias, and the lack of randomized data. Nevertheless, these clinical signals, however imperfect, provide a rationale for prioritizing the inflammatory burden hypothesis in future prospective studies, rather than serving as current validation of it.

However, these results shouldn’t be taken as proof of the proposed causal mechanism. Different drug classes may affect PCa in different ways, and factors such as a patient’s baseline inflammation, disease activity, and treatment duration could influence outcomes, leading to inconsistent conclusions. Critically, these pharmacoepidemiologic observations cannot serve as a counterargument to null MR findings; MR and pharmacoepidemiology address fundamentally different questions—the former examining lifelong genetic susceptibility, the latter reflecting treatment-related associations in specific clinical contexts. Well-designed prospective studies with stratified analyses by drug type, dosage, treatment duration, and IBD subtype are urgently needed to clarify the true impact of various treatment strategies on PCa risk. Translating these pharmacological clues into clinical action would require risk-stratified screening protocols that account for cumulative inflammatory exposure, an approach we outline in more detail in Section 7.

## 5 Methodological challenges and controversies in causal inference

### 5.1 Detection bias as a primary driver of positive observational findings

Detection bias operates through three interlocking mechanisms that inflate PCa incidence estimates in IBD populations without a true biological increase. First, ascertainment bias arises because IBD patients, particularly those with UC, undergo frequent gastroenterological surveillance, inadvertently increasing opportunities for incidental PSA testing and prostate biopsy compared to non-IBD controls. Second, inflammation-driven PSA elevation has been causally demonstrated. Li et al. showed, using both NHANES data and MR, that UC increases PSA concentrations through non-malignant pathways, thereby directly inflating biopsy referral rates (30). Third, verification bias compounds this effect, as biopsies are preferentially performed on men with elevated PSA, a phenomenon that mathematical models can correct but which is rarely accounted for in epidemiological studies (80). The fact that MR studies—which are immune to healthcare utilization confounders—consistently yield null results strongly implicates detection-related artifacts as major contributors to positive observational estimates. However, detection bias alone cannot account for all discrepancies, as it does not explain why some observational studies with similar surveillance intensities report divergent results.

### 5.2 Limitations of Mendelian randomization in capturing acquired inflammation

While MR offers a powerful tool to circumvent confounding and reverse causation, its application to the IBD–PCa question faces two overarching constraints that limit its ability to definitively refute causality. First, the generalizability of existing MR results is restricted by the predominance of European-ancestry GWAS data; transferability to East Asian, African American, and other populations with distinct IBD/PCa epidemiological profiles remains uncertain. Second, and more critically, MR captures lifelong genetic predisposition rather than the dynamic, time-varying nature of acquired inflammation. This fundamental disconnect means that MR cannot test the hypothesis that cumulative inflammatory burden acquired during adulthood—rather than inherited susceptibility—promotes prostate carcinogenesis. Null MR findings do not exclude a causal role for acquired inflammation; they merely indicate that germline genetic variants associated with IBD do not confer prostate cancer risk through the same pathways. The assumption of no horizontal pleiotropy, while statistically testable, is also inherently unprovable and may be violated in complex autoimmune–cancer relationships.

Beyond these overarching constraints, two additional factors limit the interpretability of MR findings in the IBD–PCa context. First, heterogeneity between IBD subtypes poses a substantial challenge. UC and CD are distinct disease entities with different genetic architectures, inflammatory profiles, and systemic effects. Pooling them in MR analyses may obscure subtype-specific effects, particularly if one subtype carries a stronger biological link to prostate carcinogenesis than the other. However, most existing MR studies lack the statistical power to conduct well-stratified analyses by IBD subtype, and the genetic instruments used may not capture the causal pathways relevant to each disease form equally. Second, gene–environment interactions represent a fundamental limitation of the MR framework in this context. MR assumes that the genetic variants used as instruments are independent of environmental confounders. However, the expression and functional impact of IBD-associated genetic variants may be modulated by environmental factors—such as diet, smoking, microbiome composition, and antibiotic exposure—that are themselves associated with both IBD activity and PCa risk. These interactions are not captured by standard MR approaches, which estimate an average causal effect across populations rather than accounting for context-dependent effects. Consequently, a null MR finding does not exclude the possibility that inflammation promotes prostate carcinogenesis in specific subgroups, for instance, in individuals with high cumulative inflammatory exposure, early-onset IBD, or environmental backgrounds. This underscores the need for complementary approaches, including prospective cohorts with detailed environmental and clinical phenotyping, to resolve the causal question.

Beyond these pharmacological observations, the interplay between inflammation and sex hormones warrants explicit consideration in the IBD–PCa context. IBD exhibits notable gender differences in incidence, disease phenotype, and natural history, with some evidence suggesting that females may have a milder disease course and lower risk of certain extraintestinal manifestations. These differences point to a potential immunomodulatory role of sex hormones. In parallel, PCa is an androgen-dependent malignancy, and androgen deprivation therapy (ADT), a mainstay of PCa treatment, has been shown to reduce the risk of UC, as noted above—implying that androgens may influence intestinal inflammation, albeit through incompletely understood mechanisms (37).

The concept of an inflammatory-hormonal axis is supported by several lines of indirect evidence (81, 82). In IBD, this translates into a male-predominant pattern for some disease features, although findings remain inconsistent across studies (83–85). Mechanistically, androgen receptors are expressed on various immune cells, including T cells and macrophages, and androgen signaling can modulate cytokine production, T-cell polarization, and barrier function (86–88). Furthermore, androgens influence the composition and metabolic activity of the gut microbiome, and reciprocal interactions among the microbiome, sex hormones, and systemic inflammation have been documented (47, 89). From the prostate perspective, chronic inflammation can disrupt local androgen metabolism within the prostate microenvironment, potentially altering the balance between androgen synthesis, degradation, and receptor signaling. This creates a two-way cycle. Gut inflammation may affect overall androgen levels or signaling, while androgens might influence IBD activity and its systemic effects through immune regulation (37, 90).

However, direct evidence linking the inflammatory-hormonal axis to PCa risk in IBD patients is scarce, and most inferences are drawn from animal studies or from observations in non-IBD populations. Whether the gender-related differences in IBD translate into differential PCa risk among male and female IBD patients is an important but largely unanswered question. Future studies should incorporate sex-stratified analyses, longitudinal measurements of sex hormone levels, and assessment of androgen receptor signaling to clarify whether the inflammatory-hormonal axis represents a genuine driver of PCa risk in IBD or a bystander phenomenon.

### 5.3 The overlooked role of cumulative inflammatory burden

Perhaps the most critical gap in the current evidence base is the complete absence of studies that incorporate time-updated measures of inflammatory burden as the primary exposure. In colorectal cancer, cumulative mucosal inflammation—quantified by disease duration, extent, and histological activity—is strongly associated with malignancy risk (59). By direct analogy, chronic systemic inflammation in IBD may exert a time- and dose-dependent effect on prostate carcinogenesis, yet no study has systematically captured longitudinal disease activity to quantify cumulative exposure over decades. A single-center study reported a median latency of 13 years between IBD diagnosis and first malignancy, with PCa among the most common malignancies observed, and a recent 5-year cohort analysis found that continuous inflammatory activity increases overall malignancy risk (91). The issue of latency is further compounded by the age discrepancy between IBD diagnosis and PCa onset (24, 26). Because IBD often presents decades before the typical age of PCa diagnosis, the true carcinogenic effect of cumulative inflammatory burden may only become apparent after 20–30 years of follow-up—a duration that exceeds the observation period of most existing cohorts. Failure to follow patients into the age range of highest PCa incidence may therefore systematically underestimate the true risk, particularly for patients with early-onset IBD who carry the longest potential exposure to chronic inflammation. Critically, patients in active inflammatory states exhibit elevated circulating cytokines that could activate NF-κB/AKT pathways in the prostate (15), whereas sustained remission or effective biologic therapy may attenuate this systemic spillover—as tentatively suggested by the observation of zero PCa cases among biologic-treated patients in a Japanese cohort (24). However, these are indirect clues; direct evidence linking cumulative inflammatory exposure to incident PCa is entirely lacking. This gap not only fuels the observational–MR discordance but also precludes the development of rational risk stratification strategies.

### 5.4 Insufficient research into pathogenic mechanisms

Although preliminary evidence suggests that chronic inflammation may affect the prostate microenvironment through systemic pathways, our current understanding of the molecular and cellular mechanisms by which IBD promotes prostate cancer development remains very limited. Existing mechanistic studies have largely focused on animal models or small-sample clinical observations, lacking in-depth functional validation and pathway analysis. For example, a study in a mouse model of chronic colitis found increased infiltration of CD45+ leukocytes in the prostate, accompanied by elevated levels of pro-inflammatory factors such as TIMP-1, CCL5, and CXCL1, as well as activation of the AKT and NF-κB signaling pathways, enhanced oxidative stress, and increased expression of DNA damage markers (15). Although these findings suggest that intestinal inflammation may induce the formation of a tumor-promoting microenvironment in the prostate, it remains unclear how inflammatory signals are transmitted from the distal intestine to the prostate, nor have the specific sources and target sites of key mediators been identified. Furthermore, the role of the gut microbiota in the IBD-prostate cancer axis has so far been limited to correlational analyses. Although the composition of the gut microbiota is significantly altered in IBD patients (86), and certain microbial metabolites possess systemic immunomodulatory functions (40), there is currently no direct evidence that specific microbial communities or their metabolites can migrate to the prostate and influence its carcinogenic process. It is worth noting that a cross-sectional study comparing patients with prostate cancer and benign prostatic hyperplasia did not even find significant differences in rectal microbiota composition, and explicitly excluded IBD patients (92), reflecting insufficient sample representativeness and disease specificity in mechanistic research in this field.

## 6 Future research directions and technical pathways

Three methodological challenges undermine causal inference in the IBD–PCa association: detection bias inflating observational estimates, MR’s inability to capture time-varying acquired inflammation, and the lack of studies using cumulative inflammatory burden as the primary exposure. Addressing these requires an operationalized framework that simultaneously resolves causality and delivers clinically actionable evidence.

The cornerstone is a multi-center prospective cohort of men with IBD, embedded in existing registries, with standardized PSA screening that includes fixed-interval testing, uniform biopsy referral criteria applied consistently across sites, and explicit documentation of all tests and outcomes to enable adjustment for verification bias, alongside a matched non-IBD control group undergoing identical protocols. The primary exposure is a time-updated cumulative inflammatory burden score that integrates disease extent, endoscopic activity, quarterly fecal calprotectin and serum CRP measurements, and treatment exposure, rather than a binary IBD diagnosis. This design tests whether cumulative inflammation predicts incident PCa in a dose-dependent manner, identifies risk thresholds, and assesses whether sustained remission normalizes risk.

It should be emphasized that this cumulative inflammatory burden score is a hypothetical research tool proposed for use in prospective study design, rather than an established or validated clinical instrument. Weighing its components would require empirical derivation and validation in the target population. Conceptually, we propose an initial weighting scheme based on the relative contribution of each component to systemic inflammation and colorectal cancer risk, as extrapolated from the IBD-colorectal cancer surveillance literature: disease extent, endoscopic activity, fecal calprotectin, serum CRP, and treatment exposure. These weightings could be refined through data-driven approaches, such as Cox regression or machine learning, as the prospective cohort accumulates outcomes. To date, no standardized cumulative inflammatory burden score has been validated to predict PCa risk in IBD patients. However, the proposed framework is grounded in the well-established paradigm that cumulative mucosal inflammation—quantified by disease duration, extent, and histological activity—is strongly associated with colorectal cancer risk in IBD (59). Analogous approaches have been used to predict other inflammation-related complications. The validity of this score for PCa risk prediction would be a key hypothesis to be tested in the proposed cohort, and its components would be refined iteratively as data accrue.

To move from correlation to mechanism, the cohort includes a nested case–control study with serial biospecimens to formally test five prespecified hypotheses: that pre-diagnostic serum glutamate and inflammatory cytokines discriminate future prostate cancer cases from controls and correlate with colonic FOLH1 expression and cumulative inflammatory burden; that glutamate and cytokines mediate the pathway from inflammatory burden to PCa; that gut microbiome composition, particularly Proteobacteria abundance, predicts subsequent PCa risk independently of inflammatory burden; that intestinal PSMA-PET signal correlates with endoscopic activity, FOLH1 expression, and circulating glutamate, thereby offering a non-invasive imaging biomarker; and that circulating glutamate and cytokines at concentrations observed in the cohort synergistically activate AKT/NF-κB signaling in prostate epithelial cells, as validated *in vitro* and mechanistically framed by the GCPII–glutamate–mGluR1 axis proposed in Section 3.3.

Practical challenges and feasibility considerations. While the framework outlined above represents an ideal design, its implementation would face several real-world obstacles. First, the cost of repeated PSMAPET imaging is high across most healthcare systems. Using it routinely in large-scale groups would be cost-prohibitive, which limits this kind of imaging to nested sub-studies or specific high-risk subgroups. Given the high cost and limited accessibility of PSMA-PET imaging for routine application, it is important to consider more accessible surrogate biomarkers that could serve as adjunctive tools for risk stratification in clinical practice. Among these, serum glutamate—a direct downstream product of GCPII enzymatic activity—is a promising candidate, as it is measurable by routine clinical laboratory assays and has been shown to correlate positively with Gleason score in patients with prostate cancer (67). Whether serum glutamate levels can serve as a non-invasive proxy for intestinal GCPII activity and predict PCa risk in IBD patients remains to be tested, but this hypothesis is directly testable in prospective cohorts with serial biospecimen collection. Additionally, fecal microbial signatures—such as Proteobacteria abundance or specific metabolite profiles—offer an accessible, non-invasive option, as stool samples can be collected repeatedly without specialized equipment or causing patient discomfort. While these biomarkers are currently at an investigational stage and lack prospective validation in IBD populations, their ease of measurement and low cost make them attractive candidates for large-scale screening or surveillance programs. However, their utility for clinical decision-making would require rigorous validation, including demonstration of predictive performance independent of established risk factors, before they could be incorporated into routine practice. Second, long-term follow-up over decades poses substantial risks of patient attrition, which could introduce selection bias and undermine statistical power; this would require dedicated retention strategies, regular patient engagement, and integration with existing clinical registries. Third, the technical infrastructure for multi-omics profiling, standardized biospecimen processing, and centralized PET image interpretation demands specialized equipment and trained personnel that may not be available at all participating centers, potentially limiting the generalizability of findings. Fourth, the heterogeneity of IBD phenotypes and treatment regimens across centers would necessitate complex statistical adjustments and large sample sizes to achieve adequate power for subgroup analyses. These challenges do not invalidate the proposed design, but they underscore the need for pragmatic adaptations—such as prioritizing PET imaging for high-risk subgroups, leveraging existing healthcare databases to reduce follow-up costs, and establishing multicenter consortia to pool resources and expertise—to translate this ambitious vision into a feasible research program.

This framework leverages existing infrastructure, provides clear go/no-go criteria, and offers direct translational return. If cumulative burden predicts risk, it can guide risk-stratified screening; if glutamate or microbial markers prove predictive, they become adjunctive tools; if the GCPII–glutamate–mGluR1 axis is confirmed, GCPII inhibitors already in IBD trials could be repurposed for dual benefit; if the association is solely detection bias, screening de-escalation is justified. Though ambitious, this strategy justifies the investment required to resolve the paradox and establish evidence-based guidelines for the growing male IBD population.

Clinical translation and management implications. If future prospective cohorts confirm that cumulative inflammatory burden independently predicts PCa risk, several practical consequences will follow. First, risk-stratified screening could be implemented: male IBD patients with high cumulative inflammatory exposure might benefit from earlier or more frequent PSA testing, whereas those in sustained remission could follow age-based general-population guidelines. Second, treatment decisions could incorporate cancer risk as an additional consideration: optimizing anti-inflammatory therapy to achieve deep remission may not only improve IBD outcomes but also reduce long-term PCa risk—a potential dual benefit that could be communicated to patients when discussing treatment goals. Third, patients with persistently elevated inflammatory markers despite optimal therapy might be candidates for closer urological surveillance or adjunctive chemoprevention, such as 5-ASA, although this remains investigational. Conversely, if the epidemiological signal proves predominantly attributable to detection or verification bias, screening de-escalation in IBD patients would be justified, reducing unnecessary biopsies and patient anxiety. Until these data emerge, a pragmatic approach would be to individualize screening decisions based on IBD phenotype, disease activity trajectory, and shared decision-making between gastroenterologists and urologists, rather than applying a one-size-fits-all policy.

## 7 Conclusion

The association between IBD and prostate cancer remains a paradox: observational studies consistently suggest an elevated risk, yet Mendelian randomization analyses do not support a causal genetic relationship, implicating confounding, detection bias, and differential healthcare utilization as major drivers. Mechanistically, chronic inflammation, gut dysbiosis, and the emerging GCPII molecular bridge provide a biologically plausible narrative that warrants investigation. Specifically, the proposed GCPII--glutamate--mGluR1 axis offers a coherent but entirely hypothetical molecular narrative. It must be emphasized that this axis currently lacks direct experimental validation and rests on independent, compartmentalized observations rather than on integrated evidence. Crucially, it remains unknown whether the cytokines, microbial products, or glutamate implicated in these pathways reach the prostate at concentrations sufficient to drive carcinogenesis—a gap that must be addressed before any causal inference can be made. Similarly, the pharmacoepidemiologic observations discussed are consistent with the inflammatory burden hypothesis but do not constitute evidence that anti-inflammatory therapy prevents PCa. The core unresolved question is whether the epidemiological signal reflects a true causal effect of cumulative inflammatory burden—which remains an unproven hypothesis—or is predominantly attributable to surveillance artifacts. Future efforts should harness prospective cohorts with time-updated inflammatory exposure metrics, multi-omics causal frameworks, and IBD subtype-stratified strategies to move beyond descriptive epidemiology toward clinically actionable paradigms for precision management of IBD-associated prostate cancer. At present, however, IBD-specific screening or risk stratification cannot be considered evidence-based.
